# Biomarker Case-Detection and Prediction with Potential for Functional Psychosis Screening: Development and Validation of a Model Related to Biochemistry, Sensory Neural Timing and End Organ Performance

**DOI:** 10.3389/fpsyt.2016.00048

**Published:** 2016-04-14

**Authors:** Stephanie Fryar-Williams, Jörg E. Strobel

**Affiliations:** ^1^The University of Adelaide, Adelaide, SA, Australia; ^2^Youth in Mind Research Institute, Norwood, SA, Australia; ^3^The Queen Elizabeth Hospital, Woodville, SA, Australia; ^4^Basil Hetzel Institute for Translational Health Research, Woodville, SA, Australia

**Keywords:** biomarkers, psychosis, mental illness, model, case detection, case prediction, schizophrenia, schizoaffective

## Abstract

The Mental Health Biomarker Project aimed to discover case-predictive biomarkers for functional psychosis. In a retrospective, cross-sectional study, candidate marker results from 67 highly characterized symptomatic participants were compared with results from 67 gender- and age-matched controls. Urine samples were analyzed for catecholamines, their metabolites, and hydroxylpyrolline-2-one, an oxidative stress marker. Blood samples were analyzed for vitamin and trace element cofactors of enzymes in catecholamine synthesis and metabolism pathways. Cognitive, auditory, and visual processing measures were assessed using a simple 45-min, office-based procedure. Receiver operating curve (ROC) and odds ratio analysis discovered biomarkers for deficits in folate, vitamin D and B6 and elevations in free copper to zinc ratio, catecholamines and the oxidative stress marker. Deficits were discovered in peripheral visual and auditory end-organ function, intracerebral auditory and visual processing speed and dichotic listening performance. Fifteen ROC biomarker variables were divided into five functional domains. Through a repeated ROC process, individual ROC variables, followed by domains and finally the overall 15 set model, were dichotomously scored and tallied for abnormal results upon which it was found that ≥3 out of 5 abnormal domains achieved an area under the ROC curve of 0.952 with a sensitivity of 84% and a specificity of 90%. Six additional middle ear biomarkers in a 21 biomarker set increased sensitivity to 94%. Fivefold cross-validation yielded a mean sensitivity of 85% for the 15 biomarker set. Non-parametric regression analysis confirmed that ≥3 out of 5 abnormally scored domains predicted >50% risk of caseness while 4 abnormally scored domains predicted 88% risk of caseness; 100% diagnostic certainty was reached when all 5 domains were abnormally scored. These findings require validation in prospective cohorts and other mental illness states. They have potential for case-detection, -screening, -monitoring, and -targeted personalized management. The findings unmask unmet needs within the functional psychosis condition and suggest new biological understandings of psychosis phenomenology.

## Introduction

The architecture of psychosis is largely unknown, and in everyday psychiatric practice, diagnoses are made by assessing observable signs and symptoms that are recognized as criteria for diagnoses in descriptive-based classification systems. However, clinical confidence in the utility of such descriptive diagnostic systems is waning in the face of multiple overlapping comorbidities within diagnoses. These limitations have increased the call for objective investigations to find medical models to provide diagnostic certainty and explain serious mental illness states (1, 2).

Clinicians are often confronted with complex or uncertain presentations of individuals with psychosis. Such individuals may not be substance abusing or have a brain disease, yet still feature elements of what is called functional psychosis, with some symptom features being prominent and others less prominent (3). Such presentations may cause a prolonged period of diagnostic uncertainty that is stressful for both clinician and patient alike, leading to treatment delay and trauma linked to poorer outcomes (4, 5). For these reasons, there is a pressing need to discover cost-effective, utilitarian biomarkers to identify psychosis with certainty. Such an advance would provide a great leap forward for clinicians and patients alike (6), would reduce time to treatment, and thereby assist recovery and reduce cost-care burden (7, 8). Accordingly, the Mental Health Biomarker Project formed an experimental plan to assess biochemistry, physiology, symptom, and behavioral endpoints for functional psychosis and compare outcome data with that from a control sample. The aim was to discover, quantify, and explore the association of any discovered biomarkers with functional psychosis, as mainly represented by diagnoses of schizophrenia and schizoaffective disorder. The plan was also to categorize any biomarker findings into a model to provide case detection and reduce uncertainty for diagnoses of functional psychosis.

As part of this plan, a number of candidate markers were selected for investigation of their biomarker status across a number of neurobiological levels. Indole–catecholamines and their metabolites have been extensively investigated in many body fluids, in relationship to psychosis, and there has been contrasting findings (9). These and other biochemical markers, such as vitamins and mineral enzyme cofactors and intermediate substances within these indole–catecholamine synthesis and metabolism pathways, were selected. Despite the potential of these key enzyme cofactors to exert subtle and cumulative effects on neurotransmitter synthesis and metabolism (Section S1 and Figure S1 in Supplementary Material), they have not been specifically investigated for their biomarker potential. The relationship of these cofactors to the methylation and folate cycles has been well summarized by Frankenburg (10) and in other review literature (11), including that derived from translational findings of this project (12).

Clinical experience, pilot study evidence, and implications from literature reports (13) guided the choice of candidate markers selected for auditory processing and end-organ acuity measures. Pilot study findings, literature references, and clinical experience with spatial working memory deficits (14) and working memory deficits (15) in diagnosed patients formed the basis of an interest in exploring the potential of candidate markers for visual and auditory processing disorder and delay. Reports of abnormal preattentive cortical-evoked responses in schizophrenia (16) implied that seeking biomarkers of sensory end-organ dysfunction in middle ear and visual acuity (17) might also be a promising field of biomarker investigation.

## Materials and Methods

### Study Setting and Design

We undertook a retrospective study using a case–control design to discover and quantify biomarkers for functional psychosis in schizophrenia and schizoaffective disorder. The study was approved by the Queen Elizabeth Hospital Research Ethics Committee (No: 2009139), and all protocols and methods used in the project conformed to that committee’s relevant regulatory standards. Prospective participants were screened and enrolled between May 2010 and December 2013 at a large urban hospital, the Queen Elizabeth Hospital, Woodville, SA, Australia, and two of its satellite mental health clinics in the Western Adelaide community catchment area (for further information, see Sample Characteristics). Cases were evaluated on the ward, in satellite psychiatric outpatient clinics, and some of these elected to be assessed in the nearby research institute setting where all control participants were assessed. Data relating to this manuscript was collected between May 2010 and December 2013 and the study is still ongoing. The authors report no conflict of interest at the time of undertaking this research or writing this paper. An international patent application was filed in December 2014.

### Participants

Recruitment of patients with mainly schizophrenia and schizoaffective disorder allowed sufficient numbers of patients with functional psychosis to be obtained within the confines of the multiple exclusion criteria described below. This also gave scope for exploration of correlative and predictive translational relationships between different biomarker variables.

Participants from multi-ethnic backgrounds in an age range between 18 and 60 years were enrolled in the study. All participants were informed of the goals, assessment procedures, and funding of this study. All participants provided written consent. Participants were diagnosed, screened for multiple exclusion criteria, rated for clinical and subclinical and symptoms, and had biological samples taken prior to further neurophysiological assessment.

In order to offset severity bias and to allow patients recovery time sufficient to understand the conditions of consent, non-detained hospital cases with functional psychosis were recruited and assessed in the expected last week of their admission, as judged by their expected discharge date on ward-round consensus opinion. Community cases were not assessed if they were in a known state of relapse for any reason. Some cases were diagnosed with first-episode psychosis; however, for most cases, diagnosis had been sustained and stabilized for a considerable time prior to their recruitment. Antipsychotic medication remained stable during the assessment period. DSM IV-R criteria-based case diagnoses (18) case diagnoses were made by registrars, confirmed by consultants and ward-round consensus, and separately checked by DSM IV-R symptom checklist. Individuals with features of psychosis, who fully met criteria for selection, were allowed for recruitment even if they lacked a firm diagnosis for schizophrenia or schizoaffective disorder, in which case a diagnosis of psychosis for investigation (psychosis FI) was allocated. However, the majority of cases were diagnosed with schizophrenia and schizoaffective disorder, and no difference in consensus diagnostic opinion was encountered.

Participants were screened for multiple exclusion criteria before entering the study. The aim was to impose sufficient exclusion criteria to minimize confounding variables and strip psychosis in the case sample as far as possible down to its bare functional state, in order to expose which candidate markers had strong discrimination and case-detection variables for functional psychosis. These exclusion criteria are described in detail below and relate to potential confounding factors, such as substance abuse, organic causes, and medication effects on candidate markers. Due to imposition of multiple exclusion criteria, recruitment of eligible cases for the study was slow, and a consent rate of only one in four eligible participants was found during the recruitment process. In this slow, unpredictable recruitment context, random sampling of successfully recruited cases was not undertaken. Implementation of the exclusion criteria process did, however, result in a highly characterized group of patients being enrolled in the study. Results on case recruitment and assessment are provided below and at the beginning of the Results Section “Sample Characteristics.”

Exclusion criteria included medication with clozapine and olanzapine, which are frequently prescribed medications for ward and outpatient clinic patients with repeated admissions for psychosis. Together with antihistamines, the following medications have prominent histamine-binding effects and so were excluded as histamine was a candidate biomarker. Patients taking antipsychotic agents, such as zuclopenthixol, modecate, amisulpride, quetiapine, and risperidone, were included. Persons on mood stabilizing medications were allowed. Persons with active or unremitted use of alcohol or other substance abuse were excluded, since this can confound neurotransmitter results. Persons with organic cerebral damage, as evidenced by a clinically documented, investigated, or descriptive history of hospitalized head injury, unconsciousness, or central nervous system disease, were also excluded, as were persons with upper respiratory tract infections, middle ear congestion, or known sensory disability. Persons with extrapyramidal signs in ocular, arm, or hand muscles (19) were excluded prior to consent. Persons receiving active vitamin therapy were also excluded due to the inclusion of vitamins as candidate markers. It was not possible to exclude smoking and have any chance of patient recruitment.

Control participants were age- and sex-stratified based upon patient recruitment data, and they were then recruited by the Population Research and Outcomes Studies (PROS) Unit of the University of Adelaide. Eligible participants were randomly phone recruited from participants in the North West Adelaide catchment area associated with the Department of Medicine at the Queen Elizabeth Hospital. Persons with diagnosed mental illness, disorientation, documented or anecdotal history of substance abuse, head injury, visual or hearing disability, learning disability, movement disorder, or taking antihistamine medication or vitamin supplementation similar to candidate markers were excluded. No control participant had a diagnosis of schizophrenia or any DSM-diagnosable mental illness but was rated for subclinical symptoms. Further information regarding control recruitment, characteristics, and assessment is provided at the beginning of the Results Section “Sample Characteristics.”

### Outcome Measures

All study participants were assessed in a real-world setting by a psychiatric trained assessor who was not blind to their study status. Ratings for cases were undertaken by ward registrars and consultants and for controls by a single psychiatrically trained researcher. All raters were blind to index laboratory and sensory processing test results at the time of rating. All participants were separately rated on standardized clinical measures as outlined, with citations, in Section S2 in Supplementary Material. These measures included the Clinical Global Impression of Severity (CGI), Global Assessment of Function (GAF), and Social and Occupational Functioning Assessment Scale (SOFAS). Clinical and subclinical symptoms were rated using the Brief Psychiatric Rating Scale (BPRS), which has many symptom overlaps with those within the Positive and Negative Syndrome for schizophrenia (PANSS); therefore, symptom scores were amalgamated in the interest of reducing assessment time. The symptom intensity rating (SIR) for each symptom (designated 1–7) was summated to give a symptom-intensity (SIR) index for each participant, which was taken as a measure of clinical severity. Additional outcome measures were collected for hospitalization frequency and disability pension status since these measures relate to community cost-care burden of psychosis management. Degree of stress was not specifically documented within the research protocol; however, the intensity of three BPRS/PANSS stress-related symptom ratings for anxiety, tension, and dissociation were analyzed for the strength of their Spearman’s correlates in relationship to biomarkers findings. Information was gathered on some risk predictors for schizophrenia or schizoaffective disorder, such as presence or absence of a family history of schizophrenia, depression or mania, developmental disorder (DD) history, or learning disorder (LD) history. Information was also gathered on history of ear infection and premorbid subclinical (non-concussed) head injury.

### Specimen Collection and Biochemical Assays

Candidate biochemical markers were selected for reasons broadly outlined in Section “Introduction.” Collection methods for these candidate markers are documented (with citations) in Section S3 in Supplementary Material of this manuscript. Specimens collected from blood were for assay of vitamin B6 (20, 21), vitamin B12, red cell folate and plasma homocysteine (22–28), serum copper (29), serum ceruloplasmin (30), red cell zinc (31), serum histamine (32), and serum methyltetrahydrofolate reductase (MTHFR 677 C → T) gene polymorphism, which has potential influence on transfer of methyl groups to the methylation cycle (Section S1 in Supplementary Material) *via* folate (12, 33). Vitamin D was also selected because of its epidemiological link with schizophrenia (34). Urine assay for levels of creatinine, dopamine (DA), noradrenaline (NA), adrenaline (AD), and two of their metabolites [homovanillic acid (HVA) and methoxyhydroxymandelic acid (MHMA)], as well as the serotonin metabolite 5-hydroxyindoleacetic acid (5-HIAA), were undertaken. Urine was also collected for levels of hydroxyhemopyrroline-2-one (HPL), which is a theoretical indicator of oxidative stress and disturbed porphyrin synthesis in schizophrenia (35). Urine was collected early in the morning with the patient in a fasting, rested state. The separation time between blood collection and urine collection was 2 h. As outlined in Section S3 in Supplementary Material, biological sample collection, transport, and storage was standardized by protocol, and testing of all samples was conducted by independent commercial laboratories that were blind to the case or control status of the participant.

### Sensory Processing Assessments

Sensory processing assessments were made after clinical exclusion of extrapyramidal side effects affecting vision, neck, or hand coordination (Sections S4 and S5 in Supplementary Material) (19). Visual assessments using the participant’s usual glasses were conducted after an alternate-cover-test to exclude visual fixation disparity [phoria (36)]. Assessment consisted of binocular near and distance visual acuity, visual attention span and speed, and accuracy of visual processing (Section S4 in Supplementary Material). Assessments were undertaken at a time separated from blood and urine collection by a minimum of 2 h and within a maximum time of 4 days. Assessments were performed by a single psychiatrically trained researcher. Assessors were not blinded to participant status because the residual symptoms of psychosis make this impractical; however, assessors were blind to laboratory results.

Auditory processing assessments (Section S5 in Supplementary Material) were conducted in a quiet room (ambient noise level 20 dB), preceded by examination of the external auditory meatus to exclude obvious pathology or sebum obstruction. Auditory acuity characteristics were examined between 250 and 4000 Hz to determine air-bone conduction gaps of >10 Hz or threshold shift abnormalities >1000 Hz and laterality differences. Equipment used in sensory processing testing is shown in Section S6 in Supplementary Material (Figures S2 and S3 in Supplementary Material).

Middle ear acoustic reflexes were directly measured as documented in Section S7 in Supplementary Material. Middle ear compliance and sound conductance across the middle ear chamber gave parameters for ear canal volume at threshold auditory response. Peak middle ear pressure at threshold auditory response and the gradient of the middle ear pressure are parameters that reflect the response of the tympanic muscle as it contracts as sound enters the ear. The effect of tympanic contraction is then relayed *via* a chain of ossicle bones across the middle ear to the stapes muscle. Stapes contraction is effected by the stapes muscle applying the handle of the stapes bone to the oval window of the cochlea, which has the effect of dampening sound as it enters the cochlea. The strength and timing of the stapes reflex response was directly measured in order to ascertain the alacrity or delay of this middle ear sound-dampening mechanism. Where repeated testing was necessary, an interval of 30 s or more was allowed between trials in order to prevent error from muscle fatigue.

### Statistical Analysis

Power analysis for this study (37) is outlined in Section S8 in Supplementary Material. The principals behind the processes that comprise formation of the 15 and 21 biomarker sets and their respective domains, regression analysis for these, model validation procedures, and the relationship of findings to functional outcome measures and stress-related symptoms are outlined below.

Sample characteristics analysis was conducted using XLSTAT (Addinsoft) (38) for descriptive statistics and STATA software (39). Variable distributions were also analyzed by Kolmogorov–Smirnov analysis; associations were tested using Pearson chi-square, likelihood ratio, and Fishers exact test. Independent-sample Mann–Whitney *U* test (40) was used to detect sample difference where there was lack of normal distribution in continuous candidate variables across groups. Data characteristics related to risk factors (Section S10 in Supplementary Material) and medication profile for cases were also analyzed using STATA (Section S11 in Supplementary Material).

Receiver operating characteristic analysis (41, 42) was carried out using XLSTAT software (38) in order to discover biomarker variables and examine their characteristics. With respect to the area under the ROC curve (AUC), this measure indicates an outcome variable’s ability to discriminate between cases and control outcome measures. When this discrimination is good, a curve with a large area underneath it is achieved. An AUC of 0.5–0.7 represents poor discrimination, 0.7–0.8 acceptable discrimination, 0.8–0.9 excellent discrimination, and >0.9 outstanding discrimination. A high sensitivity means that a test only rarely misses classifying a person with schizophrenia/psychosis as having such diagnosis, and therefore, the test has utility as a diagnostic method. A high specificity means that a test only rarely designates a person with schizophrenia/psychosis as being free of that diagnosis, so the test therefore has utility as a diagnostic exclusion, screening tool. Sensitivity and specificity are considered acceptable at ≥85% and ideal at ≥90%. Missing data were imputed using XLSTAT (38). Diagnostic associations for ROC variables were also analyzed for odds ratio (OR) (43)^1^, where OR ≥2 was considered important.

Discovered ROC analysis variables, their parameters, and OR values were divided into domains based upon their function. Depending upon their unique ROC analysis cutoff point, variables resulting from ROC analyzes (ROC variables) in each different functional domain were scored dichotomously for abnormality and underwent ROC analysis again to obtain a threshold abnormal ROC score for each separate functional domain. These domain scores were tallied and the ROC process repeated again to derive ROC parameters for the whole biomarker model set and to obtain a cutoff threshold of abnormality for case detection using the whole model set. In this way, ROC variables within each of the segregated domains and their cutoff ranges were combined into an overall ROC Model from which could be determined a case-detection threshold and reference standard for diagnostic accuracy. Comparison was made between missing data imputed and non-imputed data outcomes for these biomarker sets of discovered variables. Compound outcome variables of theoretical interest were also examined by ROC analysis and the theory behind their selection is outlined in Results Section “Characteristics of Residual and Compound ROC Variables.”

Regression analysis was undertaken to identify the predictive risk of schizophrenia or psychosis related to number of abnormally scored domains, using logistic regression and Lowess locally weighted non-parametric regression analysis (43).

Spearman’s correlation analysis (38) was used to investigate compound and other ROC variables of interest and to examine the contribution of stress to the results. Specifically, appraisal of the relationships of stress symptoms for anxiety, tension and dissociation to ROC variables discovered, was carried out by Spearman’s analysis.

### Model Validation

Model validation (see Model Validation) was determined by (A)–(C), below:

(A) Case discrimination strengths for individual variables and tallied sets of variables were provided by AUCs, and the accuracy index provided by XLSTAT.
(B) Fivefold cross-validation process (44, 45). In this process, cases and controls were separately randomized into five groups and biomarker set(s) built again using four fifths of the data as a “training set” to be validated against the other fifth of the data as a “test set.” Five separate iterations were undertaken where each group was used as a validation sample only once, and the other four groups were used as a calibration sample in each fold. As a result, none of the data used to construct each biomarker set were used to validate it. Then, by comparing calibration (AUC) and prediction accuracy [using STATA software (40)], with parameters derived from the original 15 biomarker set, any optimism arising from a small sample with case–control modeling can be adjusted.
(C) As an initial form of content validity for the model, Spearman’s correlation was used to identify the relationships between the domains of the model and functional measures that were external to the model parameters (see Examination of Stress-Related Symptoms in Relationship to Risk-Predictor Variables). These functional measures were for severity (SIR and CGI), disability (GAF), and cost/support burden as determined from disability support pension (DSP) requirement and hospital admission frequency.

## Results

The total North West population number for the hospital and community clinics was estimated at 22,000 (46). Imposition of multiple exclusion criteria (see Participants) restricted eligible cases to 370, and only 25% of these consented to participation. This high refusal rate resulted in 89 consenting cases, of which 7 did not reach assessment due to declining mental state and 15 were excluded due to the confounding factor of SSRI and SNRI medication. This was due to detection of an interesting masking effect of SSRI and SNRI medication on catecholamine levels, sensory processing performance, and middle ear outcome measurements. Due to the above factors, case enrollment in the study was slow; therefore, random sampling of recruited cases was not undertaken. Number of cases in the final analysis was 67, which still included 3 cases treated with a SSRI.

The control sample was drawn from an available sample number of 2489 (47). After stratification of patients for age- and sex matching with control participant extraction, randomization and imposition of recruitment exclusion criteria, and a low consent rate of 25%, a total of 72 control participants were recruited. Five of these were excluded due to failure to meet exclusion criteria on face-to-face history taking and assessment. No control participant had a diagnosis of schizophrenia or any DSM-diagnosable mental illness but was rated before assessment for reported and observed subclinical symptoms in a real-world setting, by a psychiatric trained assessor who was not blind to their study status.

### Sample Characteristics

Final data analysis was based on data from 67 cases and 67 control participants. Section S12 in Supplementary Material records the level of missing data, which was 1.5% for the laboratory-derived variables, 8.2% for the visual assessment variables, and 7.5% for the auditory assessment variables. Table 1 presents the breakdown characteristics of participants according to their gender age, diagnosis BMI, age of onset, duration of illness, and intensity of rated symptoms (SIR). Secondary tables containing data distribution analysis and chi-square analysis are included in Section S10 in Supplementary Material. Difficulty was encountered recruiting sufficient young persons to volunteer as controls; so, the mean age of the control population is 5 years older than the patient sample, and this difference was detected on independent-sample Mann–Whitney *U* test (*P* = 0.006). The Kolmogorov–Smirnov sample distribution analysis for BMI (*P* = 0.000) showed it to be non-normally distributed. An independent-sample Mann–Whitney *U* test failed to detect significant statistical difference between cases and controls for BMI.

**Table 1:** **Sample characteristics (SE)**.

Characteristic	Schizophrenia			Schizoaffective psychosis			Psychosis FI			Cases			Controls			Total participants		
	*n*	Mean	SE	*n*	Mean	SE	*n*	Mean	SE	*n*	Mean	SE	*n*	Mean	SE	*n*	Mean	SE
Age	33	40.7	2.0	30	40.8	1.9	4	37.3	7.4	67	40.5	1.3	67	45.7	1.4	134	43.1	1.0
Age of onset	31	23.9	2.0	27	22.5	1.5	3	32.7	10.1	61	23.7	1.3	0			61	23.7	1.3
Duration of illness (DOI)	31	16.5	1.9	27	18.0	2.3	3	8.0	1.0	61	16.8	1.4	0			61	16.8	1.4
Symptom intensity rating (SIR)	33	113.8	5.7	30	94.4	5.7	4	119.5	14.8	67	105.4	4.0	67	42.8	0.3	134	74.1	3.4
Body mass index (BMI)	23	31.1	2.3	27	29.6	1.4	3	25.8	1.2	53	30.0	1.2	66	26.7	0.6	119	28.2	0.6
Right hand dominance %	32	92.1	3.0	29	94.5	2.4	4	82.5	17.5	65	92.6	2.1	67	93.1	1.7	132	92.8	1.3
Urine creatinine (mmol/L)	33	8.8	0.9	29	9.3	1.2	4	12.3	5.1	66	9.2	0.7	67	9.5	0.7	133	9.3	0.5
5-Hydroxyl indole acetic acid (5-HIAA)	33	4.6	1.4	29	3.3	0.8	4	9.5	4.8	66	4.3	0.8	67	1.6	0.1	133	2.9	0.4
Plasma homocysteine (μmol/L)	33	10.5	0.4	29	9.5	0.5	4	9.3	1.4	66	10.0	0.3	66	9.5	0.3	132	9.7	0.2
Red cell acetylcholine esterase (U/gb Hb)	29	38.3	0.9	29	41.6	1.2	3	37.3	1.9	61	39.8	0.7	67	39.6	0.7	128	39.7	0.5
Hearing threshold (dB)	31	548.4	27.0	23	587.0	67.8	3	500.0	0.0	57	561.4	30.8	60	550.0	19.5	117	555.6	18.0
Visual threshold of near vision	32	6.5	0.6	26	6.0	0.7	3	8.0	3.0	61	6.3	0.5	67	5.2	0.1	128	5.7	0.2

	**Schizophrenia**			**Schizoaffective psychosis**			**Psychosis FI**			**Cases**			**Controls**			**Total participants**		
		***n***	**%**		***n***	**%**		***n***	**%**		***n***	**%**		***n***	**%**		***n***	**%**

Sex
Female		12	36.4		16	53.3		2	50.0		30	44.8		34	50.7		64	47.8
Male		21	63.6		14	46.7		2	50.0		37	55.2		33	49.3		70	52.2
Persons		33	100.0		30	100.0		4	100.0		67	100.0		67	100.0		134	100.0

Data characteristics related to risk factors for functional psychosis collected within the history protocol are also given in Section S10 in Supplementary Material. A list of psychiatric medications for participants is included in Section S11 in Supplementary Material.

### ROC and Odds Ratio Results

Fifteen biomarkers were selected firstly for their strength of discrimination on ROC analyses but also for their sensitivity or specificity (Table 2). Biomarker findings were for elevated urine levels of DA, NA, AD, and the urine oxidative stress marker hydroxypyrroline-2-one/creatinine, adjusted for creatinine levels (HPL/creatinine). Deficit-related biomarkers were discovered for red cell folate, pyridoxal-5′-phosphate coenzyme form of activated B6, and the activated 25-OH form of serum vitamin D. Serum B12 had lower discrimination capacity but higher specificity, and the ratio of percentage of free copper to red cell zinc also reached biomarker capacity. Within the sensory processing spectrum of outcome variables, significant ROC variables were discovered for visual (symbol) attention span and threshold visual speed of processing performance with respect to norm for age, expressed in terms of a per cent value. Though binocular distance vision was relatively impaired in cases versus control, outcome measures representing impaired long distance acuity for visual information from the right eye was discovered to have biomarker status. Reverse digit span (which measures auditory or verbal working memory) and speed of auditory processing, at threshold hearing level, expressed as percentage of norm for age was a further biomarker. A significant ROC variable for the competing words listening task performance (expressed as a percentage of norm results for age), is a notable result as this is a dichotic listening test that measures cross-hemispheric ability to attend to auditory information presented simultaneously to both ears.

**Table 2:** **Fifteen biomarker set with odds ratio**.

Domain name and number	ROC set No.	ROC variables	No. Obs	AUC	SENS	SPEC	PPV	NPV	% Risk of rejecting Ho	ROC *P* value	Odds ratio^+^	Odds ratio *P* value	Accuracy
1. Visual	1	Low visual span	126	0.862	0.831	0.821	0.021	0.999	0.01	<0.0001	22.46	<0.0001	0.8254
	2	High visual speed of processing discrepancy (% of age)	122	0.875	0.909	0.731	0.015	0.999	0.01	<0.0001	27.22	<0.0001	0.8115
	3	Poor distance vision on right	128	0.597	0.475	0.851	0.014	0.997	0.01	<0.0001	5.17	0.0001	0.6719
2. Auditory	4	Low reverse digit span	127	0.810	0.900	0.552	0.009	0.999	0.02	0.000	11.1	<0.0001	0.8707
	5	High competing words discrepancy (% of pass score) (represents dichotic listening disorder)	124	0.799	0.759	0.773	0.015	0.999	0.01	<0.0001	10.69	<0.0001	0.8362
	6	High auditory speed of processing discrepancy (% of age)	121	0.874	0.745	0.879	0.027	0.999	0.01	<0.0001	21.23	<0.0001	0.6810
3. Catecholamine	7	High dopamine	133	0.702	0.379	0.940	0.028	0.997	0.01	<0.0001	9.60	<0.0001	0.6617
	8	High noradrenaline	133	0.851	0.742	0.881	0.027	0.999	0.01	<0.0001	21.25	<0.0001	0.8120
	9	High adrenaline	133	0.844	0.758	0.821	0.019	0.999	0.01	<0.0001	14.32	<0.0001	0.7895
4. Oxidative stress	10	High (HPL/creatinine)^a^ (oxidative stress domain)	133	0.696	0.697	0.642	0.009	0.998	0.01	<0.0001	4.12	<0.0001	0.6692
5. Biochemistry–nutrition	11	High free copper to zinc ratio	133	0.611	0.470	0.746	0.008	0.997	2.19	0.022	2.60	0.0104	0.6090
	12	Low B6 activation	129	0.638	0.800	0.484	0.008	0.997	0.17	0.002	3.75	0.0009	0.6434
	13	Low red cell folate	133	0.654	0.591	0.716	0.009	0.997	0.10	0.001	3.64	0.0005	0.6541
	14	High serum B12 (80% CI)	134	0.565	0.373	0.761	0.007	0.996	18.56	0.186	1.89	0.0933	0.5672
	15	Low vitamin D	132	0.651	0.462	0.791	0.010	0.997	0.12	0.001	3.24	0.0026	0.6288

*Fifteeen biomarkers in five for schizophrenia and schizoaffective disorder. All values reported at 95% confidence interval unless otherwise stated*.

*ROC, receiver operating curve; No Obs, number of observations; AUC, area under receiver operating curve (ideally >0.6); SENS, sensitivity; SPEC, specificity; pre-adjusted odds ratio + prior to division by 3, *P* value for odds ratio at 95% confidence interval; % RR-Ho, percentage risk ratio for null hypothesis (ideally <0.10), HPL, urinary hydroxyhemopyrroline-2-one*.

*^a^Catecholamines did not require imputation*.

Six biomarkers were discovered on middle ear performance testing. These included biomarkers for tympanic and stapes reflex muscle contraction, threshold percentage length of the base of the stapes reflex divided by the total duration of the reflex (a measure of the strength of the stapes reflex during its maximal period of contraction), threshold stapes amplitude projected (alternative measure of stapes contraction strength), threshold time to offset of the stapes reflex contraction divided by the base length (gives a measure of acoustic reflex decay), threshold ear canal volume (as measure of middle ear integrity), threshold peak middle ear pressure, and threshold gradient middle ear pressure (related to tympanic reflex strength), as outlined in Table 3. Raw outcome measures relating to these ROC variables can be quickly measured on clinical assessment; however, this requires more expensive equipment (Section S7 in Supplementary Material), greater clinical expertise and more data transformation into meaningful measures than that required for the previously described 15 ROC variables above. Also, their addition to the 15 biomarker set did not add discrimination strength to the overall set of 21 biomarkers, though it did impart marginally higher sensitivity for psychosis risk-prediction and case-detection purposes. For these reasons, the six middle ear biomarkers are considered to have potential utility for case detection when an individual has symptoms that are highly suggestive of psychosis but their more-easily assessed 15 biomarkers do not reach threshold for case detection of functional psychosis.

**Table 3:** **Additional six middle ear ROC variables, comprising the full 21 biomarker set with odds ratio**.

Domain name and number	Set number	ROC variables	No. Obs	AUC	SENS	SPEC	PPV	NPV	% Risk of rejecting Ho	ROC *P* value	Odds ratio^a^	Odds ratio *P* value	Accuracy
6. Middle ear (supplementary domain)	16	High threshold ear canal volume	123	0.603	0.367	0.825	0.006	0.997	0.01	<0.0001	2.74	0.0181	0.6016
	17	Low threshold peak middle ear pressure	124	0.617	0.700	0.484	0.006	0.997	0.04	0.000	2.19	0.0369	0.5887
	18	High threshold gradient middle ear pressure (90%)	124	0.580	0.370	0.891	0.013	0.997	6.74	0.0674	3.77	0.0064	0.6129
	19	High threshold stapes amplitude projected	123	0.626	0.583	0.651	0.007	0.997	0.29	0.003	2.61	0.0099	0.6179
	20	Low threshold time to offset/base length	122	0.659	0.683	0.613	0.008	0.998	0.01	0.001	3.42	0.0013	0.6475
	21	High threshold percentage base length/duration	122	0.657	0.583	0.774	0.120	0.998	0.14	0.001	4.80	0.0001	0.6803

*Twenty-one biomarkers in a total of six domains for schizophrenia and schizoaffective disorder. All values reported at 95% confidence interval unless otherwise stated*.

*ROC, receiver operating curve; No Obs, number of observations; AUC, area under receiver operating curve (ideally >0.6); SENS, sensitivity; SPEC, specificity; pre-adjusted odds ratio (prior to division by 3), *P* value for odds ratio at 95% confidence interval; % RR-Ho, percentage risk ratio for null hypothesis (ideally <0.10); HPL, urinary hydroxyhemopyrroline-2-one*.

*^a^Middle ear domain and catecholamine domain did not require imputation*.

When ROC variables were dichotomously scored for abnormality against their threshold cutoff value and these scores were tallied and ROC analyzed again, a threshold of abnormality for each domain was determined. Then, in a similar manner, these domain scores were tallied and ROC analyzed for the 15 and 21 biomarker sets, respectively, to obtain a threshold number of abnormal domains required for case detection, together with their ROC parameters (Tables 2 and 3) (Section S12 in Supplementary Material). The 15 biomarker set (with 5 domains) had a specificity range of 80–96 (mean 91%) and a sensitivity range of 69–90 (mean 82%), for identification of the relevant psychosis conditions, at the 95% level of significance. ROC variables for elevated levels of NA, AD, reduced visual span, visual speed of processing variables, and competing words deficit (dichotic listening disorder) show high sensitivity and/or high specificity for the detection of schizophrenia and schizoaffective forms of functional psychosis. Auditory speed of processing variables showed both high sensitivity and high specificity.

**Figure 1:**
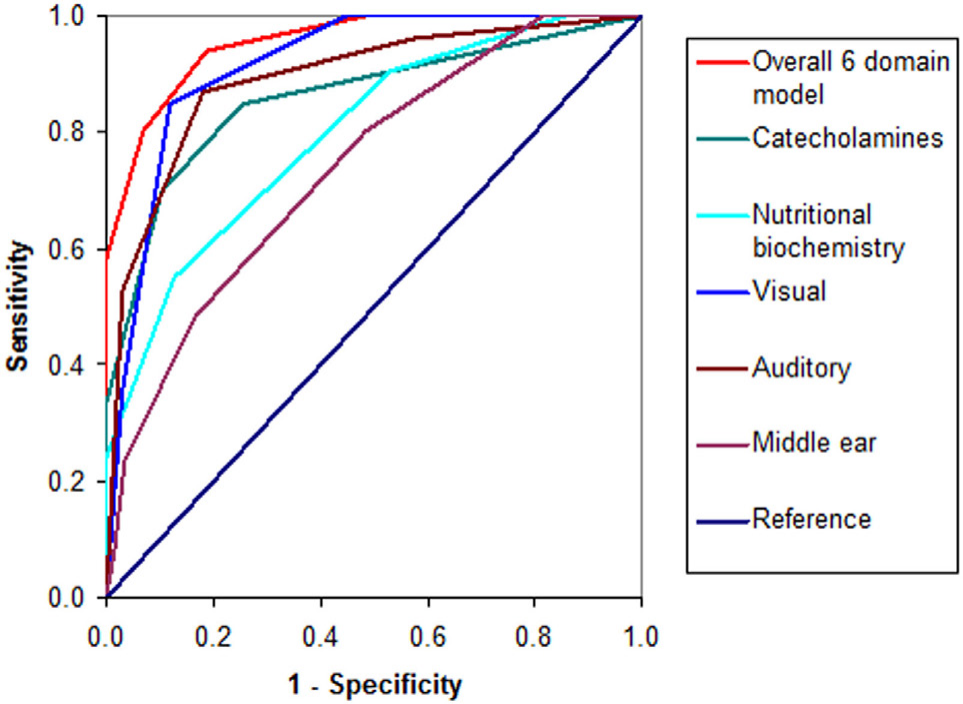
**ROC curves for the 21 biomarker set**.

The relative strengths of the functional domains represented within the 21 biomarker set are demonstrated in Figure 1.

When domain scores were tallied and the ROC process repeated again to derive a threshold of abnormality for the whole 15 biomarker (5 domains) model set (Table 4), the threshold of diagnostic detection was found to be reached at ≥3 abnormal domains. The unimputed 15 biomarker set of 5 domains had good case discrimination (AUC = 0.952) with 84% sensitivity and 90% specificity for case detection and screening purposes. This means that 15 biomarkers in 5 domains were able to identify 84% of symptomatic participants as having schizophrenia or schizoaffective psychosis and 90% of asymptomatic participants as having no schizophrenia or schizoaffective psychosis. With imputed values, the 15 biomarker set had an AUC of 0.951, a mean specificity of 91% (range of 81–96%), and a lower mean sensitivity of 82% (range of 69–90%). Its combined sensitivity and specificity was 1.836, and sensitivity was 85% on model cross-validation.

**Table 4:** **Parameters and pathological cutoff values for six functional domains of biomarkers for functional psychosis**.

Domain number and name	No. ROC variables per domain	No. Obs	AUC	SENS	SPEC	PPV	NPV	% Risk of rejecting Ho	ROC *P* value	Odds ratio	Odds ratio *P* value	Accuracy
1. Visual domain	3	120	0.915	0.849	0.881	0.031	0.999	0.010	<0.0001	41.48	<0.0001	0.8667
2. Auditory domain	3	119	0.891	0.868	0.818	0.021	0.999	0.01	<0.0001	29.57	<0.0001	0.6016
3. Catecholamine domain*	3	133	0.859	0.848	0.746	0.015	0.999	0.01	<0.0001	16.47	<0.0001	0.9790
4. Oxidative stress (HPL/creatinine)	1	133	0.696	0.697	0.642	0.009	0.998	0.01	<0.0001	4.12	<0.0001	0.6692
5. Biochemistry–nutrition	5	126	0.797	0.548	0.875	0.019	0.998	0.39	0.001	8.5	<0.0001	0.7143
Combined 15-set, 5 domain												
(Not imputed), cut off ≥3 domains	**15**	**107**	**0.952**	**0.841**	**0.905**	**0.038**	**0.999**	**0.01**	**<0.0001**	**50.21**	**<0.0001**	**0.8707**
(Not imputed) cut off ≥4 domains		**107**	**0.952**	**0.659**	**0.984**	**0.158**	**0.998**	**0.01**	**<0.0001**	**119.867**	**<0.0001**	**0.8352**
(Imputed), cut off ≥3 domains		**116**	**0.951**	**0.824**	**0.908**	**0.039**	**0.999**	**0.01**	**<0.0001**	**45.89**	**<0.0001**	**0.8362**
(Imputed), cutoff ≥4 Domains		**116**	**0.951**	**0.647**	**0.985**	**0.160**	**0.998**	**0.01**	**<0.0001**	**117.333**	**<0.0001**	
6. Middle ear domain (supplementary)*	6	120	0.738	0.48	0.520	0.130	0.997	0.03	<0.0001	4.28	0.0001	0.6583
Combined 21 set, 6 domain (imputed) with middle ear domain cutoff at 3/6 domains	**21**	**108**	**0.954**	**0.940 (0.830–0.985)**	**0.833 (0.689–0.892)**	**0.022**	**1.0**	**0.01**	**<0.0001**	**66.93**	**<0.0001**	**0.740**

*All values reported at 95% confidence interval unless otherwise stated. ROC, receiver operating curve; No Obs, number of observations; AUC, area under receiver operating curve (ideally >0.6); SENS, Sensitivity; SPEC, specificity; pre-adjusted ODDS ratio + prior to division by 3, *P* value for odds ratio at 95% confidence interval; % RR-Ho, percentage risk ratio for null hypothesis (ideally <0.10), HPL, urinary hydroxyhemopyrroline-2-one*.

*Bold font for parameters for the combined imputed and non-imputed 15 biomarker set and the combined 21 biomarker set*.

*Middle ear domain and catecholamine domain did not require imputation.

Inclusion of the middle ear biomarkers to form a set of 21 biomarkers, in 6 domains of inquiry, raised mean sensitivity to 0.94 (94%) for a range of 0.83–0.96 and identified 96% of symptomatic participants as having a diagnosis of schizophrenia or schizoaffective psychosis, with a combined sensitivity and specificity of 1.771. Despite this, the overall specificity of the imputed 21 biomarker set was only 83% (range 0.70–0.89), reducing its screening capacity. Therefore, it has supplementary utility if a diagnosis is suspected from a patient’s clinical profile, yet 15 biomarkers alone are not definitive for case detection of functional psychosis.

### Characteristics of Residual and Compound ROC Variables

A number of ROC variables were not included in the 15 and 21 biomarker sets.

ROC-related parameters of three compound variables did not add more strength to discrimination, sensitivity, specificity, or predictive parameters within the 15 and 21 biomarker sets selected. These compound variables were for DA × 5HIAA (*n* = 133, Spearman’s rho = 0.420, *P* = 0.000, OR = 1.00, *P* = 0.000, AUC = 0.745 *P* < 0.0001), NA/MHMA (*n* = 133, Spearman’s rho = 0.50, *P* = 0.000, OR = 1.00, *P* = 0.00, AUC = 0.7915, *P* < 0.0001), and AD/MHMA (*n* = 130, rho = 0.495, *P* = 0.000, *n* = 133, OR = 1.92, *P* = 0.000, AUC = 0.786, *P* < 0.0001). Nevertheless, high correlations of these compound biomarkers with caseness cannot be ignored in terms of their possible dynamic implications within the nutritional biochemistry under investigation and their potential meaning is discussed in Section “Biochemistry and Nutrition.”

**Figure 2:**
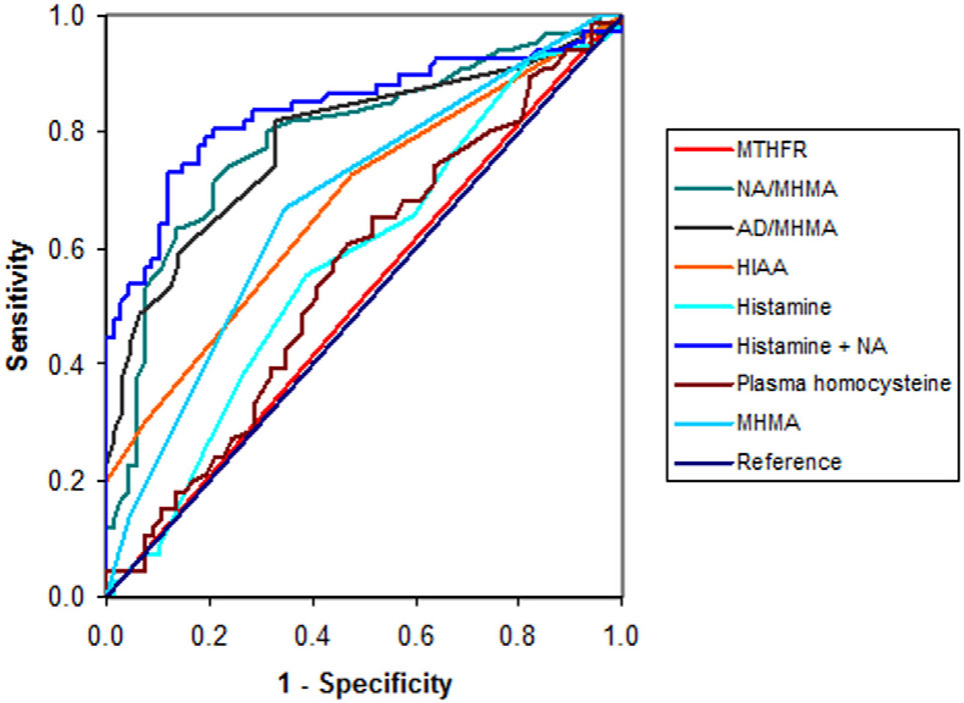
**ROC curves for variables residual to the 21 biomarker set**.

Though histamine + elevated noradrenalin (*n* = 134, Spearman’s rho = 0.59, *P* = 0.000, OR = 1.13, *P* = 0.000, AUC = 0.837, *P* < 0.0001) produced a standout AUC, this was not better than that for the single NA biomarker and histamine biomarker, alone (see Figure 2), which only produced a low discrimination ROC (*n* = 133, AUC = 0.576, *P* < 0.0001). Other selected markers that produced ROCs with poor discrimination capacity (AUC > 0.5, but <0.6, Figure 2) were for MTHFR C667T polymorphism (*n* = 134, AUC = 0.5109, *P* = 0.000), plasma homocysteine (*n* = 132, AUC = 0.557, *P* = 0.049, at 75% confidence level), and MHMA (*n* = 133, AUC = 0.678, *P* = 0.000, at 65% level of confidence). It was, however, notable that the elevated NA biomarker correlated highly to high NA/MHMA biomarker (*n* = 133, rho = 0.673, *P* = 0.0000) on Spearman’s correlation analysis, as did the AD biomarker with the AD/MHMA biomarker (n130, rho = 0.712, *P* = 0.0000). The serotonin metabolite, 5-hydroxyindoleacetic acid (5-HIAA) ROC (*n* = 133, AUC = 0.677, *P* = 0.000), was only significant at 70% confidence level, despite its independent-sample Mann–Whitney *U* test detecting a distribution difference between cases and controls (see ROC and Odds Ratio Results). Other sensory variables which failed to produce ROC discrimination capacity were outcome measures for auditory gap detection and auditory-figure ground tests within the SCAN test for auditory processing disorder and outcomes for near vision using the Sussex near vision test (Section S4 in Supplementary Material).

**Figure 3:**
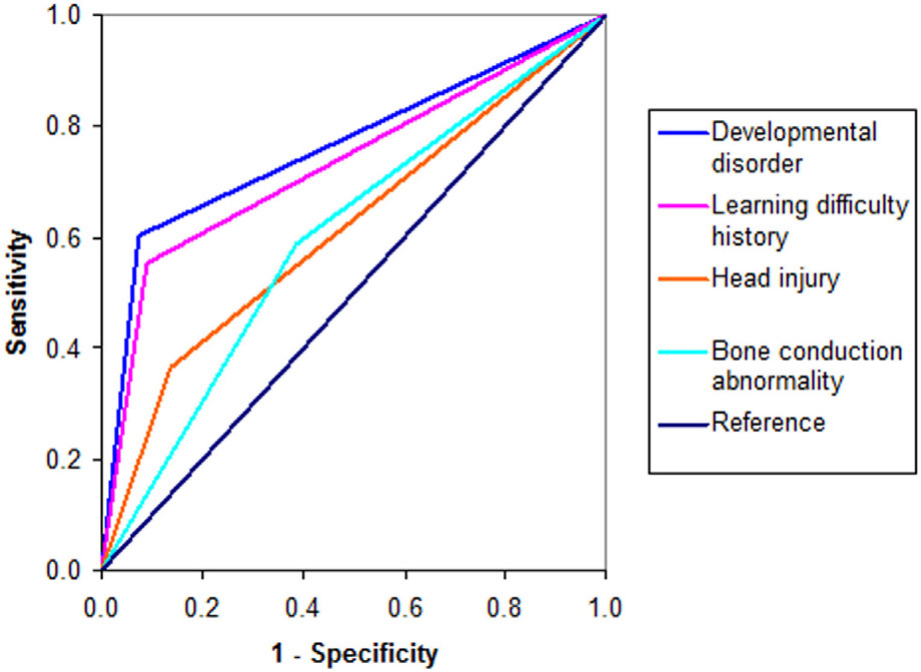
**ROC curves for risk factors**.

Collateral information gathered from participants on presence or absence of history for some risk predictors for schizophrenia or schizoaffective disorder were dichotomously scored and those that yielded significant case-discrimination ability were as follows: developmental disorder (DD) history (*n* = 103, AUC = 0.764, *P* = 0.000), LD history (*n* = 13,434, AUC = 0.731, *P* = 0.000), and subclinical premorbid head injury (CHI) (*n* = 129, AUC = 0.614, *P* = 0.001). Though a clinical history of ear infection did not yield a significant ROC and participants with significant auditory conduction disorder were excluded from participation, audiogram score for subclinical air/bone conduction discrepancy indicated that subclinical inner ear pathology is a factor of significance in functional psychosis (*n* = 128, AUC = 0.601, *P* = 0.010) (Figure 3). Given that a family history of mental illness was not an exclusion factor for recruitment of controls in our study, the fact that a positive family history yielded a low, insignificant AUC (*n* = 130, AUC = 0.539, *P* = 0.086) for our sample population is not surprising but highlights the significance of our other ROC findings. We also noted (Section S10 in Supplementary Material) that the ROC variable derived from scoring for inflammatory or sclerosis signs on tympanic membrane otoscopy was 80% related to the diagnosis of schizoaffective psychosis. Furthermore, subclinical premorbid head injury or a premorbid history of abnormal otoscopy or abnormal bone conduction findings were 100% related to the diagnosis of functional psychosis for investigation (psychosis FI) – this being a diagnostic category allocated to three patients in the study who met criteria for exclusion factors, but for whom no discrete psychosis diagnosis had been assigned.

### Regression Analysis to Determine Case-Detection Threshold and Predictive Risk

Logistic and non-parametric regression results for the number of abnormally scored domains are shown in Table 5. This analysis confirmed that ≥3 abnormally scored domains predicted (>50%) risk of case-detection threshold for the 15 biomarker set, while 4 abnormally scored domains predicted 88% risk of diagnosis and 100% certainty of diagnosis was reached when all 5 domains were abnormally scored.

**Table 5:** **Logistic and non-parametric regression for number of abnormally scored functional domains within the 15 biomarker set**.

Scored number of abnormal functional domains	0	1	2	3	4	5
% Predicted risk on logistic regression analysis	0.97	6.13	30.32	74.37	95.08	99.23
% Predicted risk on non-parametric (Lowess) regression analysis	0	3.7	38.88	66.22	88.48	100

*Logistic regression *R*^2^ (Nagelkerke) = 0.752 and non-parametric (Lowess) regression *R*^2^ = 0.626*.

### Model Validation

Biomarker set validation procedures are described in Materials and Methods Section “Model Validation” and results are presented in sequence of method.

Case–control biomarker discrimination capacity (AUC in Table 4) for the 15 biomarker set was high, as evidenced by an AUC value of 0.952 associated with an accuracy index of 87%. The 21 biomarker set had an AUC of 0.95 and an accuracy index of 74%. Comparison between case-detection performance for data imputed and non-imputed data sets also revealed minimal difference (Section S12 in Supplementary Material).

Comparison of fivefold cross-validation parameters, with the original 15 biomarker set, demonstrated maintenance of 15 set parameters for AUC, sensitivity, and specificity (Table 6). Moreover, the validity of these parameters was confirmed across a range of clinically useful schizophrenia population prevalence levels. This process provided the 15 biomarker set with a higher sensitivity value of between 85 and 92%. This process demonstrated that the original tallied 15 biomarker set is stable, and its regression results are not unduly optimistic.

**Table 6:** **Fivefold cross-validation results**.

Fold	No. Obs	Population prevalence parameters	% Sensitivity	% Specificity	ROC area	Odds ratio	% PPV*	% NPV*
1	28	0.35	78.6	92.9	0.857	47.7	3.7	99.9
		30	78.6	92.9	0.857	47.7	82.5	91.0
2	26	0.35	84.6	92.3	0.855	66	3.7	99.9
		30	84.6	92.3	0.885	66	82.5	93.3
3	28	0.35	85.7	64.3	0.750	10.8	0.8	99.9
		30	85.7	64.3	0.750	10.8	50.7	91.3
4	26	0.35	92.3	92.3	0.923	144	4.0	100.0
		30	92.3	92.3	0.923	144	83.7	96.6
5	26	0.35	84.6	92.3	0.885	66	3.7	99.9
		30	84.6	92.3	0.885	66	82.5	93.3
Scored 15 biomarker set	134	0.35	85.1	86.6	0.858	36.7	2.2	99.9
		30	85.1	86.6	0.858	36.7	73.1	93.1

*Results are reported across prevalence values that may be clinically encountered, 0.35% prevalence = mean prevalence for schizophrenia (applicable to screening) within the Australian population, where 12 monthly MEAN schizophrenia prevalence is estimated as equivalent to 0.35/100 (0.35%), 30% prevalence = estimated prevalence rate among a clinical population (where expected conversion rate to schizophrenia is 30–35% at 1-year follow-up) (89)*.

*Percent positive predictive value and negative predictive value.

When the individual ROC variables were appraised against functional rating data using Spearman’s correlation matrix (Table 7), particularly high correlates were demonstrated for a number of biomarkers in relationship to outcome measures for severity (CGI and SIR), disability (GAF and SOFAS), and cost-care burden (disability pension requirement and hospital admission rate), at 95% level of significance. Notably, strong correlates were found between abnormalities in the visual and auditory domains and disability, social dysfunction, hospitalization rate, disability support requirement, and severity measures.

**Table 7:** **Spearman correlations (in descending order of strength), for 15 and 21 biomarker sets and their functional domains, with separate functional measures of disability and severity**.

Domains	(5 domains) 15 biomarker set (imputed)	Visual	Auditory	High catecholamine	Biochemistry–nutrition	Oxidative stress (HPL/creatinine)	Middle ear	(6 domains) 21 biomarker set (imputed)
Case versus control 525	0.770	0.730	0.650	0.598	0.458	0.339	0.340	0.775
Symptom intensity rating (SIR) for psychosis	0.697	0.624	0.583	0.467	0.404	0.327	0.377	0.636
SOFAS ROC	0.752	0.729	0.632	0.591	0.415	0.312	0.341	0.758
GAF ROC	0.770	0.745	0.618	0.562	0.415	0.315	0.328	0.742
CGI ROC	0.754	0.729	0.636	0.591	0.415	0.312	0.341	0.761
Hospital admission rate	0.830	0.766	0.608	0.583	0.403	0.421	0.300	0.748
Disability pension requirement	0.677	0.608	0.530	0.460	0.309	0.296	0.212	0.609

**Model domain**		**Case versus control**	**SOFAS ROC**	**GAF ROC**	**CGI ROC**	**Hospital admission rate**	**Disability pension**	**Symptom intensity rating (SIR)**

15 biomarker set (5 domains)		0.770	0.752	0.770	0.754	0.830	0.677	0.697
Visual domain		0.650	0.632	0.618	0.636	0.608	0.530	0.624
Auditory domain		0.650	0.632	0.618	0.636	0.608	0.530	0.583
High catecholamines		0.598	0.591	0.562	0.591	0.583	0.460	0.4677
Biochemistry–nutrition domain		0.458	0.415	0.415	0.415	0.403	0.309	0.404
Middle ear domain		0.340	0.341	0.328	0.341	0.300	0.212	0.377
Oxidative stress (HPL/creatinine)		0.339	0.312	0.315	0.312	0.421	0.296	0.327
21 biomarker set (6 domains)		0.775	0.758	0.742	0.761	0.748	0.609	0.636

*All rho values are at 95% level of confidence. For rho ≥0.350, P ≤ 0.0001. SOFAS, Social and Occupational Functioning Assessment Scale; GAF, Global Assessment of Function Scale; CGI, Clinical Global Index (of Severity). Hospital admission rate as determined by number of admissions divided by duration of illness and disability pension receipt, as indices of cost burden*.

### Examination of Stress-Related Symptoms in Relationship to Risk-Predictor Variables

Spearman’s correlates were examined in relationship to stress-related SIRs for anxiety, tension, and dissociation symptoms (see Statistical Analysis). The highest correlate strength for the symptom of anxiety was found for deficits in auditory processing domains of dysfunction [auditory domain (imputed) (*n* = 224, rho = 0.553, *P* = 0.000)]. For the symptom of tension, highest correlate strength was obtained for deficits in both auditory domain (*n* = 124, rho = 0.409, *P* = 0.000) and high catecholamine domain (*n* = 133, rho = 0.408, *P* = 0.000).

Low competing words score (as a % of pass score) is an index of dichotic listening disorder. Symptoms of dissociation, such as experiencing “blank periods,” and “unreal feelings,” held interesting highest correlates for this particular ROC variable. The dissociative symptom of experiencing “blank periods” also correlated to low competing word score (*n* = 124, rho = 0.210, *P* = 0.019) in a setting where it also held significant correlates for the auditory and visual domains (imputed). In this context, the dissociative symptom of experiencing “unreal feelings” also correlated to both low competing word score (*n* = 124, rho = 0.212, *P* = 0.018) and with significant correlates in auditory and biochemical nutrition domains. There was a negative correlation between experiencing “unreal feelings” and histamine levels (*n* = 134, rho = −0.230, *P* = 0.004), which is further discussed below.

## Discussion

Outcome measures that form the primary set of 15 biomarkers in this study are derived from easily procurable laboratory tests, inexpensive equipment, and simple-to-apply assessment methods that can be conducted in a 45-min consultation in an everyday clinical setting with low ambient noise.

The 21 biomarker set brings together an accumulation of ROC-defined biomarkers from several domains of biological and neurophysiological logical inquiry. It includes biomarkers for oxidative stress (HPL/creatinine) and nutrition-related biochemistry with intra- and extracerebral visual and auditory function markers.

Though internally validated, the utility of these findings has several caveats. First, substance abuse is a common concurrent feature of psychosis presentations in the clinical context; therefore, these assessments are valid in the setting of a non-active drug use history and ideally, a negative drug screen result. Second, these results require validation in larger samples, including other mental illness states, at other sites.

Discovered biomarkers represent a number of discrete sensory disorders, such as dichotic listening disorder, sensory processing delay disorders, distance visual acuity disorder, and inner ear dysfunction. Though it would therefore be remarkable if such discrete, subclinical disorders did not occupy at least a part of the etiological substructure of the condition that is recognized at its clinical surface as functional psychosis, drawing meaning from a biomarker cannot be automatically assumed. Indeed, meaningful explanation and predictive modeling are two different goals of research and biomarkers are mathematically derived parameters which of themselves cannot denote an etiological meaning for the condition they detect. Therefore, the following discussion of the meaning and dynamics of some of the biomarkers discovered in this study is supplied for reader’s interest, with further explanations to be found in previous study reports (12).

### Elevated Catecholamines and the Meaning of Compound Biomarker Results

ROC analysis-derived cutoff values for elevated DA, NA, and AD levels indicated that an excess state of these catecholamines was highly discriminative for functional psychosis. In keeping with other literature concerning DA and noradrenergic activity in schizophrenia, these findings may reflect stress-activated hypothalamic–pituitary–adrenal (HPA) axis activation in psychosis (48–54).

In this study, we were not able to access serotonin level assays as these are only available under strict conditions related to diagnosis of serotonin-releasing gastrointestinal tumors. However, our interest in exploring the relationship of DA to serotonin’s metabolite 5HIAA is because serotonin (5HT) and DA have somewhat reciprocal roles in the brain (55). In this regard, these biomarker results for DA × 5HIAA, seemingly indicate that elevated DA in functional psychosis is exacerbated by low serotonin, such as might occur with excess serotonin metabolism, and accompanying increased excretion of 5-HIAA. Yet because the discrimination capacity of this combined biomarker (DA × 5HIAA) did not exceed that for elevated DA alone, it was not fitted into the model. It can, nevertheless, be viewed as an interesting finding, pointing toward further dynamics at work within the biochemical framework of schizophrenia. That said, the actual reliability of 5HIAA itself as an isolated marker separate from a serotonin level or a serotonin precursor l-tryptophan level must be considered dubious. Since serotonin is synthesized in the gastrointestinal tract, its levels may vary with diet. Theoretical uncertainty about the meaning of 5-HIAA also arises because of the diversion of the serotonin precursor molecule l-tryptophan away from serotonin synthesis and into the kynurenate pathway, under inflammatory conditions that have also been implicated in schizophrenia (56). Since we did not have resources to co-investigate inflammatory markers in this study and our urine samples for kynurenic pathway metabolites have not yet been assayed, we are not in a position to comment in any reliable manner on the possible dynamic significance of the compound (DA × 5-HIAA) biomarker discovery, except to say that in the absence of inflammation, it may reflect a conjoint tendency toward elevated DA levels and increased serotonin metabolism and excretion in a study setting where both DA and serotonin metabolites (HVA and 5HIAA, respectively) were found to form ROCs of low yet valid significance. In a setting where research literature reports that the stimulating effect of cortically elevated DA relies on serotonin for a contrasting dampening effect (55), high serotonin metabolism with related low serum serotonin levels might help to explain the overstimulating effects of DA within the cerebral cortex.

Since MHMA is the direct metabolite of noradrenalin, the significant spare ROC findings of NA/MHMA and AD/MHMA and their high correlative relationships to NA and AD in this study imply that metabolism of NA and AD is compromised in this sample of patients with diagnoses representative of functional psychosis (see Model Validation and biochemistry map in Section S1 in Supplementary Material). Metabolism of NA and AD is carried out by the enzyme monoamine oxidase (MAO); so, it can be surmised that MAO activity is compromised in our patient sample, a finding which is supported by literature accounts of this enzyme’s inhibition in schizophrenia and schizoaffective disorder (57).

Findings of low folate [a contributor to methylation and *S*-adenosylmethinine (SAMe) formation], together with elevated (NA and histamine), carry the implication that one-carbon cycle methylation is compromised in our sample of patients. SAMe unavailability with *S*-adenosylhomocysteine (SAH) elevation inhibits catechol-*O*-methyltransferase (COMT) catecholamine metabolism, providing an explanation for the elevated catecholamine findings in this study (Section S1 in Supplementary Material). This explanation is supported by the study finding of a low, significant ROC curve for histamine, implying that SAMe might be lower than normal and SAH higher than normal within our patient sample. This occurs because SAMe is a required cofactor for histamine metabolism by histamine *N*-methyltransferase (HNMT), and SAH also inhibits this reaction. Therefore, low global methylation may contribute to backed-up, unmetabolized, and elevated catecholamines in our patient sample. In an attempt to offset this situation, NA can also act as a methyl donor for histamine metabolism by HNMT, explaining why high serum histamine may be an unreliable surrogate marker for a low methylation state (22, 32, 58, 59).

Histamine has neurotransmitter properties that cause activation within the brain (60), while NA causes arousal, stress, and anxiety, and AD promotes fear and flight (61). It, therefore, seems either directly or indirectly because of low enzyme activity or through cofactor deficits that MAO inhibition, COMT inhibition, and inhibited histamine metabolism may together contribute to anxiety, fear, and over-arousal in our patient sample (22, 32, 57–60).

### Relationship of Stress and Psychosis to Sensory Timing and End-Organ Dysfunction

Due to past literature emphasis on the role of emotional stress in schizophrenia (62) and reported elevated catecholamines in schizophrenia and stress-related conditions (50–54), the finding of elevated catecholamines in this study led to cross-examination of stress-related symptoms of anxiety, tension, and dissociation in relationship to our ROC biomarker variables (see Statistical Analysis).

The particular finding that anxiety symptom intensity correlates more strongly to ROC biomarkers for auditory and visual domains of dysfunction than with elevated level of noradrenalin or adrenalin levels *per se*, supports our theory that disturbed auditory and visual pathway transmission may itself serve as an intracerebral interoceptive cue that precipitates HPA activation (63), followed by heightened catecholamine synthesis and release. Indeed, our previously reported Lowess regression modeling confirmed a 76% predictive relationship between visual domain biomarkers and catecholamine elevation (12). Such regression modeling also revealed that high threshold stapes amplitude was 79% predictive of longer stapes contraction duration, implying that an over-strong, over-brisk NA-driven stapes stress-response has potential to over-dampen and delay sound as it enters the cochlear. This effect may thus play a role in dampening and delaying sound transference into the brain. The brain’s subliminal recognition of such disturbed neural timing may then trigger an HPA initiated vicious cycle of further catecholamine synthesis and release (64). Though a moderate levels of NA encourage the brain to greater processing efficiency, when NA levels are gradually ramped up (or kindled) in this way, they eventually exert a detrimental effect on cortical sensory processing, resulting in sensory disconnection and a psychotic break (12, 65, 66).

It is also notable that sensory end-organ dysfunction from middle ear and long distance vision abnormalities were discovered to contribute to the dysfunctional substrate of the 15 biomarker – 5 domain set that formed the basis for regression analysis. Indeed, it is relatively easy to speculate that such visual and auditory impairments could well lead to anxiety and paranoid suspicion upon the distant approach of an unknown person in community and clinical settings. Such findings highlight the critical importance of optimal patient access to specialist services for eye and ear assessment, so that visual and auditory end-organ dysfunction can be detected and remediated early in the course of the illness in order to curtail their contribution to fear and the catecholamine kindling process that leads on to sensory circuit disconnectivity and psychosis onset (12, 67).

Our stress-related findings (see Examination of Stress-Related Symptoms in Relationship to Risk-Predictor Variables) indicate that stress itself is a heterogeneous entity that takes different forms in psychosis according to the individual profile of dysfunction within each individual’s bio-neurophysiological substrate. In anxiety, interoceptively cued sensory processing disorder predominates, but with tension, elevated catecholamines predominate.

With dissociative symptoms however, there are contrasting negative correlates for histamine levels occurring in the absence of any correlates for elevated catecholamines. This latter effect may be explained by high methylation with inversely low histamine (68), in which case we theorize that high SAMe levels and over-active COMT-related catecholamine metabolism leads to depleted catecholamine levels that challenge adrenal capacity for resynthesis resulting in reduced catecholamine release and so-called “adrenal fatigue” syndrome (69).

### Dichotic Listening Disorder, Stress, and Functional Psychosis

Dichotic listening disorder, as evidenced by high competing words discrepancy (Section S5 in Supplementary Material) (expressed as percent of normative score for age), was a notable biomarker within the 15 biomarker set (*n* = 124, AUC = 0.799, OR = 10.7, for *P* < 0.0001). Dichotic listening disorder has a known relationship to metabolic damage to the corpus callosum circuits that is related to hypoconnectivity and schizophrenia (70–72), and this intracerebral condition may be a strong target for remediation in schizophrenia and schizoaffective disorder.

### Biochemistry and Nutrition

Stress-related, HPA-triggered increase in catecholamine synthesis can draw down on l-DOPA synthesis, which utilizes cofactor vitamin B6 that is also required for glutathione synthesis and its prevention of oxidative stress (Section S1 in Supplementary Material). As previously explained, deficits in precursor folate reserves can reduce downstream SAMe production, and low SAMe and higher SAH levels inhibit COMT activation and raises catecholamine levels further. Catecholamines, in turn, lead to excessive draw down on vitamin D reserves required to offset their effects on parathyroid hormone (12, 73). Undoubtedly, maternal dietary deficiency and dietary deficiency that accompanies the chaotic lifestyle of psychosis contribute to this effect (74, 75). This is, however, a wide and complex research area that requires further consideration and longitudinal therapeutic trials designed to resolve how readily cognitive and sensory processing deficits can be reversed by targeted supplement therapy (76). Certainly, it is within the clinical experience of this author that psychosis and affective states can be considerably ameliorated by including carefully targeted adjunctive biochemistry supplements in a personalized treatment approach. Emerging reports in the research literature also attest to possible benefits from methyl folate supplementation to boost methylation (77). Beneficial use of *n*-acetyl cysteine for treatment of oxidative stress is also reported (78, 79).

### Limitations

Methodologies for discovering novel biomarkers and presenting these in models of disease pathophysiology remain in evolution and all bring a new point of view that should be globally considered (42). Though case–control study designs have inherent susceptibility to prevalence and selection bias (80), they are allowable for discovery and diagnostic accuracy studies (81) and have a respectable record when used to detect low prevalence disorders, such as schizophrenia (82). Participant refusal rate of 25% for patients and controls alike may have created sufficient random effect to offset selection bias.

The impact of the exclusion criteria on the number of subjects in the study also brings into question the broader applicability of the model, particularly with regard to particular medication exclusions and substance use exclusion (83). However, it is envisaged that the model’s use is less likely to be required for patients already established on Clozapine or Olanzapine and is more likely to be used to for early diagnostic confirmation or population screening.

Despite our attempts to isolate SSRIs from the study sample, three patients had their data analyzed while still retained on these medications. However, this would make our findings even more significant, since the overall effect of SSRIs and SNRIs was to mask elevated catecholamine levels, sensory processing deficits, and abnormal middle ear outcome measurements. Both sodium valproate and quetiapine medication were not disallowed in this study and these agents have both been associated with high catecholamine levels in animal studies (84, 85). In the case of valproate, we theorize that sodium valproate promotes GABA synthesis from excess glutamate left over from NMDA hypofunction that occurs in response action in GABA promotion, which mops up excess glutamate related to NMDA hypofunction that occurs in a reciprocal response to elevated DA levels (86). Quetiapine is a second-generation antipsychotic that has affinity for DA type 2 receptors, histamine type 1 receptors, and alpha 1 and 5HT receptors, related to anxiety inhibition. Since NA stimulates alpha-1 receptors (87) and quetiapine blocks these receptors, it is theoretically possible that quetiapine and other DA-blocking antipsychotic medications that bring about their antipsychotic effects through receptor blockade might collaterally increase residual volume transmission of catecholamines in the extracellular cerebral space (88). If this is so, then elevated histamine levels residual to histamine receptor blockade by quetiapine might also be explicable through such volume transmission dynamics.

Scoring and collapsing the data through multiple ROC analyses and later regression analysis have potential for loss of useful information and some data strength. Disease prevalence value was also set low in formulating the model, resulting in a low PPV values. Fortunately, this effect was found to be unimportant on fivefold cross-validation. Odds ratio calculations made in the context of a cross-sectional case–control design have been reported to yield results that may be inflated up to three times by prevalence bias (89). In this study, OR calculations endured even after the shrinkage factor of 3 had been applied to adjust for this possibility.

As mentioned in Section “Characteristics of Residual and Compound ROC Variables,” the selection of biomarker combinations, such as DA × 5HIAA, NA/MHMA, and histamine + elevated noradrenalin, was based upon their imputed meaning within the nutritional biochemistry pathways of interest (see Dichotic Listening Disorder, Stress, and Functional Psychosis). Though support for the etiological significance of biomarkers in this study can be drawn from previous global translational findings (12) and implied from the number of biomarkers that represent discrete brain disorders within the model itself, a mathematical case-detection model does not possess inherent content validity.

A further limitation of the case–control discovery design is that the relevance of discovered biomarkers to other forms of mental illness is unknown. Therefore the optimal means for replicating and validating this project’s discovery biomarkers is *via* a large, well-characterized, prospective, and multi-site clinic trial on a series of consecutive patients with other mental illness diagnoses. Blinded assessors and an emphasis on collecting longitudinal data from ultra high risk for psychosis medication-naive subjects as well as for participants with a variety of other mental illness is required. Issues of the effect of chaotic lifestyle and traumatic stress arising from, or embedded within, the psychosis condition itself also require further research.

## Conclusion

These findings have screening and case-confirmation potential for conditions, such as schizophrenia and schizoaffective psychosis, as a diagnosis of functional psychosis can be predicted from the results of a 45-min clinic consultation using easily accessed laboratory tests and simple-to-apply equipment.

Combining 21 biomarkers derived from outcome measures from well-characterized patients and controls into clinically accessible biomarker sets allows better statistical outcomes than using single biomarkers alone. These biomarker sets contain markers representative of several domains of biological, cognitive, and neurophysiological inquiry – in particular for elevated catecholamines, oxidative stress, nutritional and catecholamine-related biochemistry, visual and auditory processing, and visual end-organ sensory function. The unimputed 15 biomarker set has good case discrimination with 84% sensitivity and 90% specificity for case detection and screening purposes. The 21 biomarker set containing 6 additional parameters of middle ear dysfunction, has increased sensitivity for case confirmation. Therefore, the model has potential use in settings where psychosis is suspected, yet 15 biomarkers alone do not provide sufficiently strong results for case risk or case certainty.

Though predictive case capacity for biomarkers cannot be directly equated with etiology, many of the biomarkers discovered represent discrete potentially remediable biological vulnerability factors lying undetected within the fabric of functional psychosis. In particular, dichotic listening disorder may be a strong target for remediation in schizophrenia and schizoaffective disorder. Also, subclinical sensory end-organ dysfunction that has often been viewed as a mere comorbidity appears to be integral to the psychosis process itself. Such findings have potential to steer clinicians in the direction of objective testing, increase rigor of clinical assessments, and provide access for patients to specialist services in order to correct unmet needs. In due course, such changes might positively influence patient outcomes by assisting case monitoring, relapse prevention, treatment-resistant management, and judiciously sequenced, targeted treatment.

Implications from correlative findings for biomarkers with different forms of stress-related symptoms unveil a heterogeneous mix of sensory end-organ dysfunction and biochemically damaged, pre-aged sensory processing circuits. These pathologies are thought to trigger catecholamine elevation in an escalating kindling process, culminating in different forms of anxiety with disconnectivity, dissociation, and psychosis.

Ratings for disability and severity that correlate strongly to biomarkers in the model imply that targeted treatment of these biomarkers has potential to reduce hospitalization rate, disability, severity of symptoms, and cost-care burden for families, care providers, and the community alike. Such findings require further research and invite a new understanding of the biological fabric and phenomenology of functional psychosis.

## Author Contributions

SF-W: MBBS, BSc (Biochem/Pharmacol), FRANZCP (Child and Adolescent Psychiatrist) is an Honorary visiting research fellow of the University of Adelaide (2010–2016). As chief researcher, she conceived, initiated, and planned the project, lodged the Ethics application, selected all the candidate markers, designed the research protocols and research plan with stated intent to perform ROC analysis, and selected biomarkers for model formation with regression analysis for risk performance of discovered biomarkers. She oriented staff and raters in ward and clinic settings, and guided the recruitment of case and control participants, enrolled some participants, entered the raw data set, performed initial data transformations, and provided normative data, equations, and directions for later data transformations and prevalence estimates. She gave informed guidance and research plan-based feedback on the direction of statistical analysis, ROC analysis, and biomarker model formation. In particular, she undertook ROC variable selection and co-fitting for the model, and suggested and requested ROC domain summation for abnormality and full model re-ROC analysis in order to obtain a threshold for case detection, odds ratio analysis, and ROC model updating for prevalence. She also requested correlation matrix analysis of variables, functional, biological, and neurophysiological ROC variables, internal validation and cross-validation, predictive case risk and certainty analysis, and correlation of biomarkers in relationship to symptoms of stress. She interpreted data analysis within biochemical, neurological, and psychiatric theory; formatted and placed data analysis values in all tables of the manuscript; and wrote the paper. JS, MD (Bonn) Spec Psych, Psychotherapy FRANZCP, as a previous Clinical Director of Psychiatry at the Queen Elizabeth Hospital, Woodville, SA, Australia, supervised and facilitated the laboratory, ward, and community organization and coordination of components of the project, revised and reviewed the final paper for publication. Both authors read and approved the final manuscript.

## Conflict of Interest Statement

Ethics permission for the study was obtained from the Queen Elizabeth Hospital Research Ethics Committee (No: 2009139), and all protocols and methods used in the project conformed to that committee’s relevant regulatory standards. The authors report no conflict of interest at the time of undertaking this research or writing this paper. The authors alone are responsible for the content and writing of the paper. An international patent application was filed in December 2014.

## Footnotes

^1^Available from: http://easycalculation.com/statistics/odds-ratio.php
