# Supplementary material for: Biomarker Case-Detection and Prediction with Potential for Functional Psychosis Screening: Development and Validation of a Model Related to Biochemistry, Sensory Neural Timing and End Organ Performance
Source: Front Psychiatry. 2016 Apr 14;7:48. doi: 10.3389/fpsyt.2016.00048 (PMC4830821; doi:10.3389/fpsyt.2016.00048)
Supplement: Supplementary file 1 [file Presentation_1.pdf]

Supplementary Material

Biomarker case-detection and prediction with potential for functional psychosis screening: development and validation of a model related to biochemistry, sensory neural timing and end-organ performance.

Stephanie Fryar-Williams[1,2,3,4.], **Jörg E Strobel[1]** .

[1]The University of Adelaide, Adelaide, SA, Australia.
[2]The Queen Elizabeth Hospital, Woodville, SA, Australia.
[3]Basil Hetzel Institute for Translational Health Research, Woodville, SA.
[4]Youth in Mind Research Institute, Norwood, SA, Australia.

*** Correspondence:** Stephanie Fryar-Williams MBBS BSc. FRANZCP.
Honorary Research Fellow University of Adelaide, Director Youth in Mind Research Institute
106 The Parade, Norwood, South Australia, 5067. Phone +61 411310449. E mail
sfwilliams@senet.com.au

Supplementary Sections S1-S13.

Methods S1-S8
S1. Map of relevant biochemical pathways with legend
S2. Severity and disability outcome measures, with citations.
S3. Methods for candidate biochemical markers, including equipment and citations.
S4. Methods for visual assessments, including equipment citations.
S5. Methods for auditory assessments, including equipment citations.
S6. Equipment used in sensory processing testing (Figures 2, 3).
S7. Methods for middle ear assessments including equipment and citations.

Results S8-S16
S8. Power analysis.
S9. Supplementary sample characteristics data. Significance tests for sex. (Section 3.2).
S10. Data characteristics related to risk factors for functional psychosis.
S11 Medication profile for cases (STATA analysis).
S 12. Missing data imputation table.
S13 Five-fold cross validation set-up.
S14. Spare and compound biomarkers of interest.

S1. Map of Relevant Biochemical Pathways with Legend

Legend S 1 Figure 1, (in alphabetical order).

ENZYMES: BHMT - Betainehomocysteine methyltrasferase,**COMT**- catechol-o-methyl- transferase, **CBS** - Cystathione Beta Synthetase,**MAT** - Methionine adenosyltransferase, **MTHFR-** Methylenetetrahydrofolate reductase**, SAMe** - S-denosylmethionine, **MT** - Methyltransferase, **SAHH** - S-Adenosylhomocysteine-hydrolase, **MSR** - Methionine sulphoxide reductase, **MS** - Methionine synthase.
Vitamin cofactors: vitamin B6 (pyridoxine), vitamin B12 (cobalamin), vitamin C, folic acid, 5 methyl tetrahydrofolate.
Mineral enzyme cofactors: Free copper (Cu), zinc.
INTERMEDIATE SUBSTRATES: BH4 - tetrahydrobiopterin **BH2 –** dihydrobiopterin**, DMG** - Dimethylglycine, **DOPAL** –dihydroxyphenylacetaldehyde, **DOPAC - d**ihydroxyphenylacetic acid, **DOPEGAL** – dihydroxyphenylglycolaldehyde, **DOMA** – dihydroxymandelic acid **DHPG** – dihydroxyphenylglycal, **DOPA –** dihydroxyphenylalanine, **FAD -** flavin adenine dinucleotide, **5HIAA – 5**-hydroxyondolacetic acid, **HVA** – homovanillic acid, **MAO**- monoamineoxidase., **MHMA** - 3-methoxy-4-hydroxymandelic acid, **MHPG** -4-hydroxy-3-methoxyphenylglycol, **SAH-** S-adenosylhomocysteine, **TMG** - Trimethylglycine, **VMA**-Vanillylmandelic acid. **HPL** Urinary hydroxyhemopyrroline-2-one.

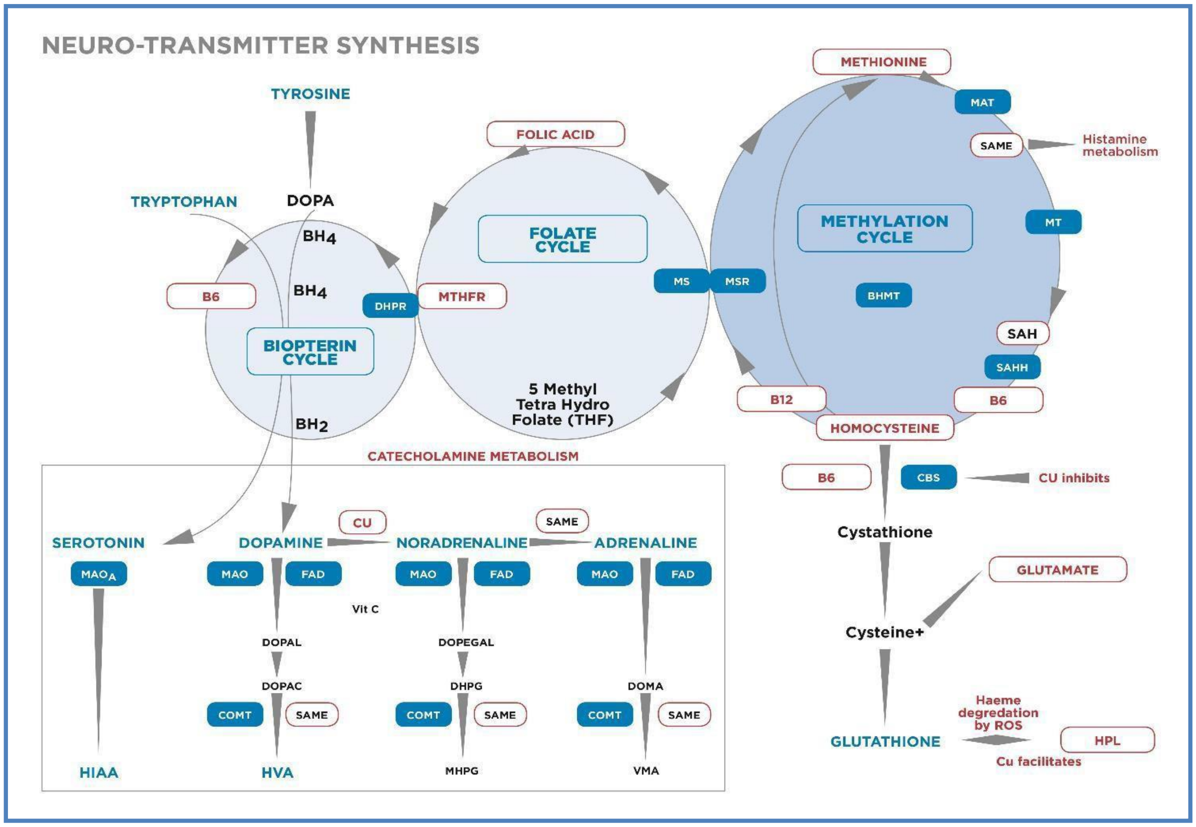

S2. Severity and disability outcome measures, with citations.

Functional Rating Scale	Citation
The Brief Psychiatric Rating Scale (BPRS)	Overall JE and Gorham DR (1962) The Brief Psychiatric Rating Scale. *Psychological Report* **10**(3): 799-812.
Positive and Negative Syndrome Scale for schizophrenia (PANSS)	Kay SR, Fiszbein A and Opler LA (1987) The positive and negative syndrome scale (PANSS) for schizophrenia. *Schizophrenia Bulletin* 13(2): 261-276.
Clinical Global Impression of Severity (CGI)	Guy W (1976) Clinical global impressions [CGI]. *ECDEU assessment manual for psychopharmacology*. National Institute of Mental Health, Early Clinical Drug Evaluation, Psychopharmacology Research Branch, Rev. Rockville, MD, U.S.
Global assessment of Function (GAF)	Frances A, Incus HA and First MB (1994) *Diagnostic and Statistical Manual of Mental Disorders*, 4th edition, American Psychiatric Association, Washington, DC.
Social and Occupational Functioning Assessment Scale (SOFAS)	Goldman HH, Skodol AE and Lave TR (1992) Revising axis V for DSM-IV: a review of measures of social functioning. *American Journal Psychiatry* **149**(9): 1148-1156.

S 3. Methods for candidate biochemical markers, including equipment and citations.

Test	Method, Analyzer, Reagents.	Laboratory/Reference
**Neurotransmitters**		
Biogenic amines: Dopamine, Noradrenaline and Adrenaline,	Spot-baseline (fasting) urinary neurotransmitter testing (second void morning), snap-frozen to minus 30 degrees and analysed by mass spectrometry, using nanomols per millimol of urinary creatinine as a standard.	SA Pathology, Adelaide, South Australia.<br><br>Whiting MJ. 2009. Simultaneous measurement of urine metanephrines and catecholamines by liquid chromatography with tandem mass spectrometric detection. *Annals of Clinical Biochemistry*, **46**:129-136
Creatinine	Spot urine specimen from the same void as biogenic amines, expressed in (millimols per Litre)	SA Pathology, Adelaide SA.
**Oxidative stress: Urinary hydroxyhemopyrro**	**Fasting urine sample collected whilst patient at rest, separated from blood drawing by minimum of 2 hours.**	
Urinary hydroxyhemopyrroline -2-one (HPL)	Spot urine (second void morning) in ascorbic acid, snap frozen (−30C) and light-protected. Colorimetric method at 540nm, following solvent extraction and reaction with Erich’s reagent. (micrograms per decilitre).	Applied Analytical Laboratories, 8/26 Nestor Drive, Meadowbrook, Queensland, Australia. +61 7 3133 1615.

S 3. Continued: Methods for candidate biochemistry markers, including equipment and citations.

Neuro-Biochemistry	All fasting blood samples, collected between 9 and 11 am daily. By arrangement, transported directly to the laboratory with no storage.	Laboratory/Reference
Vitamin D (25-OH)	Diasorin Liason assay kit, for use on the Liaison platform. (nmol/L)	Clinpath Laboratories, 19 Fullarton Rd, Kent Town. South Australia 5067 +61 8 8366 2000.
Serum total Vitamin B12	Competitive Electrochemiluminescent Immunoassay. Roche Modular E 170 Automated Immunoassay Analyser, using Roche Vitamin B12 Reagent. (nmol/L)	Clinpath Laboratories. As above.
Plasma Red Cell Folate	Competitive Electrochemiluminescent Protein Binding Assay, using Roche Modular E 170, using Roche Folate Red Blood Cell (RBC) Reagent and Roche Folate RBC Haemolysing Reagent on Automated Immunoassay Analyser. (nmol/L)	Clinpath Laboratories. As above.
Serum Vitamin B6 (Pyridoxal- 5'-phosphate coenzyme form)	Whole blood High Pressure Liquid Chromatography with fluorescent detection. Chromsystems Vitamin B6 in Whole Blood High pressure Liqid Chromatography Reagent Kit. Waters Alliance 2695 Separations Module. Waters 474 Fluorescence Detector.(nmol/L)	Sullivan Nicolaides Pathology 143 Whitmore St, Taringa. Queensland 4068. Australia. +61 7 337 8666
Serum Copper	Flame Atomic Absorption Spectrophotometry. Varian AA-240FS. (umol/L)	Douglass Hanly Moir Pathology 14 Griffnock Avenue, Macquarie Park. New South Wales 2113. +61 2 9855 5222.
Plasma Red Cell Zinc	Inductively coupled plasma mass spectroscopy	Sullivan Nicolaides Pathology.
Serum Ceruloplasmin	Immunoturbidimetric method, using 6K91-30	Douglass Hanly Moir Pathology.

Percentage Free Copper/Red Cell Zinc	Percentage of free copper in the serum calculated by an equation based on the molecular and atomic weights of ceruloplasmin and copper (one ceruloplasmin molecule binds to six copper atoms). The ratio of the percentage free copper to red cell zinc was calculated as "percentage free copper" / "Red cell zinc umol/L".	Calculated by authors
**Intermediate substrates and enzymes**		
MTHFR Ala222Val (C677T) methyl tetrahydrofolate reductase polymorphism	Real time PCR analysis Roche Diagnostics Light-Cycler 480 kit. Using TecnoBiol reagents, Sigma probes and primers on Roche LC480 analyser.	Douglass Hanly Moir Pathology.
Plasma homocysteine	Ice transported EDTA sample. Competitive Chemiluminescent Immunoassay, using Seimens Homocysteine reagent on Seimens Advia centaur Automated Immunoassay.(umol/L)	SA Pathology. +61 8 8222 3000
Serum histamine	Beckman Coulter Radio Immunoassay, using Beckman Coulter R.I.A. Kit on Perkin Elmer Wizard 1470 Automated Gamma Counter. (umol/L)	Sullivan and Nicolaides.

S4. Methods for visual assessments, including equipment citations.

Assay/Assessment	Method	Laboratory/Reference
Visual		
Near vision acuity test	Sussex Vision test of near vision. Near vision test card SNT-3000-L, 2009–2011.	Sussex Vision International Ltd. (35) http://sussexvision.co.uk/index.php/near-tests/reading-tests.html
Visual (symbol) span	Increasing number of symbols are presented in a standardised order from left to right. Test score reported as the absolute number of visual symbols recalled in the correct order.	Based on Visual Symbol Span subset test of WMS-IV (Weschler 2009) Wechsler Memory Scale - WMS-IV A&NZ Language Adapted Edition) https://www.pearsonclinical.com.au/products/view/212
Distance vision **(**Binocular distance vision acuity)	Right distance vision, then left distance vision, with 20 seconds inter-test interval.	The Snellen-Chart (Snellen 1860)
Threshold visual speed of processing performance as a percentage of age (Expresses visual processing speed in terms of the visual processing system's relative age) Shortest interval of time a person can notice between the order of presentation of two optical stimuli. Speed of visual order processing increases with age. For adults between the range of 18 and 60 years, the normal range for visual speed of processing is 24 to 72 milliseconds). For adults between the range of 18 and 60 years, the normal range for visual speed of processing is 24 to 72 milliseconds.	Person tested sees two brief flashes of light randomly presented from left-to-right or right-to-left on multiple occasions, and must decide which light flash appeared first. The inter-stimulus time interval (ISI) between the flashes is shortened by computer algorithm, if the answer is correct, otherwise it is lengthened. A performance-age rating, is provided, configured against norms-for-age. Performance-age is subtracted from the individual's actual age and the result divided by the age of the test subject is multiplied by 100.	Brain Boy Universal Professional instrument (MediTECH 2010) http://www.meditech.de/fileadmin/download/anleitungen/manual_BUP-neu-03.03.2010.indd-mail.pdf *Medi*TECH® Electronic GmbH Langer Acker 7 D-30900 Wedemark http://www.meditech.de Telefon: +49-(0)5130) 97778-0 Fax: +49-(0)5130) 97778-22 Email: service@meditech.de

S 5. Methods for auditory assessments, including equipment citations.

Assay/Assessment	Method	Laboratory/Reference
**Auditory**		
Reverse digit span (Measures auditory (verbal) working memory) Normal range is 6 to 7	With gaze aversion by listening participant and tester, digits are read in set sequence. The tested participant is asked to repeat them in reverse order. Reported as the absolute number of digits correctly recalled in reverse order.[12]	Subset of Wechsler Adult Intelligence Scale III (Wechsler 1997) Pearson http://www.pearsonclinical.com/psychology/products/100000243/wechsler-adult-intelligence-scale--third-edition-wais-iii.html
Competing words performance for age as a percentage of age (Intra-cerebral dichotic listening performance for processing of auditory information) Normal ranges vary with age	A voice-over CD and earphones test ability to correctly identify both of two competing-words (CW), delivered separately to the right and left ears. Using this test's normative-for-age database, the difference between each test subject's expected and actual performance-for-age was calculated, and this was then divided by the actual age of the test subject, and multiplied by 100.	SCAN-3:A Tests for Auditory Processing Disorders in Adolescents and Adults (Keith 2009). https://www.pearsonclinical.com.au/products/view/315
Threshold speed of Auditory processing as (Speed of auditory processing systems relative to age)<br><br>Speed of auditory processing reduces with age. For adults in the age range of 18 and 60 years, the normal range for auditory speed of processing is 46 to 72 milliseconds.	Person tested hears two clicks, randomly presented from right to left and left to right side, presented through headphones. By pressing a right or left button, a decision must be made from which side the dual-stimulus originates. If the answer is correct, the inter-stimulus interval between flashes (ISI) is shortened, otherwise it is lengthened. The auditory order threshold is the shortest ISI a person can correctly differentiate between two auditory impressions. A read-out of the threshold speed of auditory (order) processing is provided, along with a norm performance-age rating. Auditory speed of (order) processing performance as a percentage of age is calculated by subtracting the norm-for-age from the performance-age, divided by the age of the test subject, multiplied by 100.	Brain Boy Universal Professional instrument (MediTECH 2010)

S 6. Equipment used in sensory processing testing

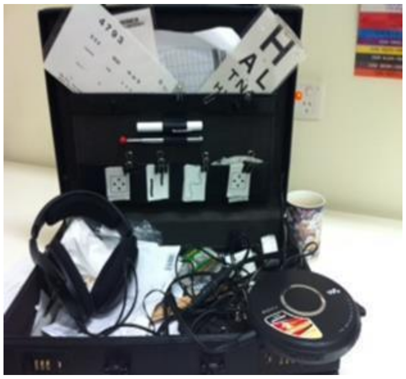

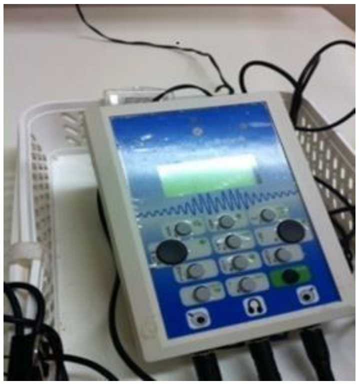

S 7. Methods for middle ear assessments including equipment and citations.

Middle ear assessments	Method	Reference
Auditory acuity assessment	Audiogram assessment	Maico Diagnostics (2005) Operating Instructions MA 40, Diagnostic GmbH, Salzufer 13/14 D-10583, Berlin, Germany. Available from: www.audiometrics.net/resources/MA40E.pdf
Ear canal volume at threshold auditory response.<br>Normal range > 0.2 cms^3	The GSI 38 measures combined volume of the ear canal and the middle ear space. An ear canal volume greater than 0.2 cm3 may indicate tympanic membrane perforation due to trauma or abnormal laxity of the tympanic membrane in response to acoustic pressure.	GSI 38 Auto Tymp Tympanometer Grason-Stadler 2005- Auto Tymp. Graason-Stadler – A division of VIASYS Health Care, 5225. Verona Rd, Building 2, Madison, WI 53711.<br>http://www.grason-stadler.com/contact-us<br>Also available from AIM Instrumentation Ltd.<br>http://aimtechnologies.ca/buy-online/attachment/gsi- 38/
Peak middle ear pressure at threshold auditory response Normal range - 150 daPa to + 100 daPa	The GSI 38 measures peak middle ear pressure achieved in response to tympanic membrane change associated with tympanic muscle contraction. This is tested over the range of +200 daPa to -400 daPa. Low pressures may accompany Eustachian tube dysfunction.	GSI 38<br>(Grason-Stadler 2005)

Gradient middle ear pressure at threshold auditory response.<br>Normal range 60 -150 daPa	The GSI 38 measures the middle ear pressure gradient, for which the normal range is 50 to 110 daPa. High gradients are indicative of middle ear effusion.	GSI 38<br>(Grason-Stadler 2005).
Percentage length of the base of the stapes reflex divided by the total duration of the reflex.<br>(A measure of the strength of the stapes reflex during its maximal period of contraction)	The GSI 38 traces the stapes reflex contraction to maximum amplitude of 8 millimetres, after which it traces a basal threshold formed for the maximum portion of the reflex. The length of this basal portion divided by the total duration of the reflex contraction was multiplied by 100.<br>(The term "threshold" refers to the first reflex response which typically occurs at a frequency of 500Hz in a decibel range of 90 to 110).	GSI 38<br>(Grason-Stadler 2005).
Projected Stapes amplitude (Alternative measure of stapes contraction strength)	Direct measurement of stapes amplitude at the intersection of projected onset and offset contraction gradients.	GSI 38<br>(Grason-Stadler 2005).
Time-to-off-set of the stapes reflex contraction divided by the base length (a measure of any acoustic-reflex offset, advance or delay).	Threshold base directly measured and compared with the total duration of the stapes reflex, from its initiation to its time of offset.	GSI 38<br>(Grason-Stadler 2005)

S8. Power Analysis. Power calculation is less relevant when calculating a sample number for case- predictive models, since there is no one specific appropriate test for a multi-variable setting where the primary goal is to obtain a stable model for regression analysis. Nevertheless, an attempt at preliminary power analysis was made, since candidate markers for some of the biochemical pathways had yielded promising results in a prior, in-clinic, case series pilot study (2007-2009). In this study, 10 out of 15 cases with schizophrenia (67%, P 0.002) and 7 out of 8 cases with auditory processing disorder (87%, P0.0002) had accumulated three or more abnormal biochemistry markers, when compared with 15 in-clinic selected control cases. These abnormal markers were for homocysteine, hydroxyhaempyrroline- 2-one (HPL), folate, zinc, percent free copper and histamine. From the sensitivity and specificity of these 6 abnormal biochemistry variables, with calculation set for 95% confidence level, a statistician calculated the need for a minimum number of 60 cases and 60 matched controls, in order to achieve power for a phase II case-control study.

S9. Secondary tables containing data distribution analysis and Chi-square analysis.

Chi-Square significance test for sex	Value	df	Asymp. Sig. (2-sided)
Pearson Chi-Square	1.877[a]	2	.391
Likelihood Ratio	1.887	2	.389
N of Valid Cases	67		

a. 2 cells (33.3%) have expected count less than 5. The minimum expected

Chi-Square significance test for sex	Value	df	Asymp. Sig. (2-sided)	Exact Sig. (2-sided)	Exact Sig. (1-sided)
Pearson Chi-Square	.479[a]	1	.489		
Continuity Correction[b]	*.269*	1	.604		
Likelihood Ratio	.479	1	.489		
N of valid cases	134			.604	.302

a. 0 cells (0.0%) have expected count less than 5. The minimum expected count is 32.00.

b. Computed only for a 2x2 table

Fishers exact test. N of valid cases

S10. Data characteristics related to risk factors for functional psychosis.

		Schizophrenia		Schizoaffective psychosis		Psychosis FI	
		n	%	n	%	n	%
FAMILY HISTORY	No	20	66.7%	16	55.2%	3	75.0%
	Yes	10	33.3%	13	44.8%	1	25.0%
DEVELOPMENTAL DELAY	No	9	29.0%	14	50.0%	2	50.0%
	Yes	22	71.0%	14	50.0%	2	50.0%
LEARNING DISORDER HISTORY	No	12	36.4%	16	53.3%	2	50.0%
	Yes	21	63.6%	14	46.7%	2	50.0%
HEAD INJURY (subclinical)	No	21	70.0%	15	51.7%	4	100.0%
	Yes	9	30.0%	14	48.3%		
EAR INFECTION	No	18	62.1%	18	69.2%	4	100.0%
	Yes	11	37.9%	8	30.8%		
OTOSCOPY	No	23	71.9%	21	80.8%	2	100.0%
	Yes	9	28.1%	5	19.2%		
BONE CONDUCTION	No	11	34.4%	12	46.2%	2	66.7%
	Yes	21	65.6%	14	53.8%	1	33.3%

S11. Medication profile for cases (STATA analysis.

	Condition type			
	Schizophrenia	Schizoaffective	Other	Total
ABILIFY	1	1	0	2
AMISULPRIDE	1	1	0	2
CHLORPROMAZINE	1	1	0	2
FLUPENTHIXOL	1	1	0	2
HALOPERIDOL	0	1	0	1
LITHIUM	0	3	0	3
BENZODIAZEPINE	2	2	0	4
PALIPERIDONE	1	0	0	1
QUETIAPINE	2	8	0	10
RISPERIDONE	18	7	3	28
VALPROATE	2	2	0	4
ZIPRAZIDONE	1	1	0	2
ZUCLOPENTHIXOL	4	1	0	5
MODECATE	0	1	0	1
OTHER	1	9	1	11
Total	35	39	4	78
Cases	29	28	4	61
Valid cases:	61			
Missing cases:	6			

S12. Missing data imputation table.

Domain type	Observed	Missing	Total	% data missing
Laboratory-derived variables	126	2	134	1.5
Visual variables	120	11	134	8.2
Auditory variables	120	10	134	7.5

S 13. 5 fold cross-validation set-up (based on AUC values).

Imputed values, 50% prevalence, for 95% CI.	5 fold cross-validation groups					Original 15 biomarker set.
**Variable**	**1**	**2**	**3**	**4**	**5**	
	**AUC**	**AUC**	**AUC**	**AUC**	**AUC**	**AUC**
Low Visual span	0.723	0.730	0.845	0.829	0.880	0.862
High Visual speed of processing discrepancy (% of age)	0.655	0.757	0.846	0.853	0.874	0.875
Poor Distance vision on right	0.617	0.456	0.623	0.591	0.608	0.597
Low Reverse digit span	0.646	0.710	0.798	0.767	0.801	0.810
High Auditory speed of processing discrepancy (% of age)	0.609	0.801	0.883	0.875	0.894	0.874
High Competing words discrepancy (% of pass score)	0.728	0.644	0.801	0.763	0.774	0.799
High Dopamine	0.661	0.679	0.700	0.694	0.715	0.702
High Noradrenaline	0.781	0.817	0.832	0.817	0.887	0.851
High Adrenaline	0.777	0.823	0.818	0.824	0.869	0.844
High Free copper to Zinc ratio	0.591	0.626	0.598	0.596	0.581	0.611
Low B6 activation	0.683	0.607	0.662	0.593	0.612	0.638
Low Red cell folate	0.530	0.628	0.696	0.662	0.635	0.654
High Serum B12	0.573	0.534	0.548	0.608	0.557	0.565
Low Vitamin D	0.521	0.670	0.667	0.632	0.690	0.651
High HPL/Creatinine)	0.648	0.757	0.704	0.695	0.652	0.696
**15 biomarkers tallied (NOT imputed)**						

S14. Spare and compound biomarkers of interest.

	XLSTAT				Stata			
	**n**	**AUC**	**SE**	**p**	**n**	**AUC**	**SE**	**p**
MTHFR homozygous polymorphism	134	0.5109	0.0000.		134	0.5109	0.0445	0.4032
NA_MHMA	133	0.7915	0.0386	< 0.0001	133	0.7915	0.0402	0.0000
HIAA	133	0.6774	0.0000.		133	0.6774	0.0427	0.0000
Histamine	134	0.5755	0.0000.		134	0.5755	0.0493	0.0628
Histamine NA	134	0.8389	0.0355	< 0.0001	134	0.8389	0.0363	0.0000
Plasma HCY	132	0.5574	0.0491	0.1213	132	0.5574	0.0502	0.1264
Serum B12	134	0.5650	0.0491	0.0928	134	0.5650	0.0497	0.0955
MHMA	133	0.6776	0.0000 .		133	0.6776	0.0420	0.0000
AD_MHMA	130	0.7865	0.0000 .	<0.0001	130	0.7865	0.0402	0.0000
DA/HVA	133	0.5828	0.0488	0.0450	133	0.5828	0.0494	0.0469
